# Endogenous Catecholamine Release in COVID-19 Related Acute Respiratory Distress Syndrome: Link between Enhanced Sympathetic Stimulation, Cardiac Dysfunction and Outcome

**DOI:** 10.3390/jcm12041557

**Published:** 2023-02-16

**Authors:** Valentino Dammassa, Marta Voltini, Costanza Natalia Julia Colombo, Gilda Maria Siano, Claudia Lo Coco, Vittoria Rizzo, Francesco Corradi, Francesco Mojoli, Guido Tavazzi

**Affiliations:** 1 Experimental Medicine, University of Pavia, 27100 Pavia, Italy; 2 Adult Intensive Care Unit, Royal Brompton Hospital, London SW3 6NP, UK; 3 Unit of Anesthesia and Intensive Care, Department of Clinical-Surgical, Diagnostic and Pediatric Sciences, University of Pavia, 27100 Pavia, Italy; 4 Anesthesia and Intensive Care, Fondazione IRCCS Policlinico San Matteo, 27100 Pavia, Italy; 5 Clinical Chemistry Laboratory, Department of Molecular Medicine, Fondazione IRCCS Policlinico San Matteo, 27100 Pavia, Italy; 6 Department of Surgical, Medical and Molecular Pathology and Critical Care Medicine, University of Pisa, 56126 Pisa, Italy

**Keywords:** COVID-19, SARS-CoV-2, catecholamines, acute respiratory distress syndrome, inflammation, echocardiography

## Abstract

The aim of this study was to measure the serum levels of catecholamines in patients admitted to intensive care unit (ICU) with COVID-19-related acute respiratory distress syndrome (ARDS) and describe their relation with clinical, inflammatory and echocardiographic parameters. Serum levels of endogenous catecholamines (norepinephrine, epinephrine and dopamine) were measured at ICU admission. We enrolled 71 patients consecutively admitted to ICU due to moderate to severe ARDS. 11 patients (15.5%) died during the admission in ICU. Serum levels of endogenous catecholamines were significantly elevated. Norepinephrine levels were higher in those with RV and LV systolic dysfunction, higher CRP, and higher IL-6. Patients with higher mortality rate were those with norepinephrine values ≥ 3124 ng/mL, CRP ≥ 17.2 mg/dL and IL-6 ≥ 102 pg/mL. Univariable analysis by Cox proportional hazards regression modelling showed that norepinephrine, IL-6 and CRP had the highest risk of acute mortality. Multivariable analysis showed that only norepinephrine and IL-6 retained in the model. Marked increase of serum catecholamine levels is present during acute phase of critically ill COVID-19 and it is associated with inflammatory and clinical parameters.

## 1. Introduction

COVID-19 is still posing a worldwide medical challenge despite the advances in understanding of its pathophysiology. A key pathogenic role has been attributed to SARS-CoV2-induced systemic inflammatory response accompanied by immune dysregulation and cytokine storm. Acute stress response with activation of the hypothalamic-pituitary-adrenal axis has been studied evidencing a correlation between serum cortisol levels and mortality [1]. The effect of increased endogenous catecholamine levels is associated with cardiovascular dysfunction in stress cardiomyopathies [2].

It has been proposed that catecholamines, especially in excess concentrations, cause a dysregulation of the physiological cascades which may be closely linked to adverse outcomes in COVID-19. However, the role of catecholamines in COVID-19 pathophysiology has not been investigated yet [3].

We described the acute catecholamine plasma concentrations measured in patients admitted to intensive care unit (ICU) with COVID-19-related acute respiratory distress syndrome (ARDS).

## 2. Materials and Methods

A cohort of patients consecutively admitted to ICU due to moderate to severe ARDS caused by SARS-CoV-2 infection (defined as positive RT-PCR from a nasopharyngeal swab and/or bronchoalveolar lavage or sputum) was enrolled.

The serum levels of endogenous catecholamines (norepinephrine, epinephrine and dopamine) were measured with high performance chromatography (Chromosystem GmbH^®^) at ICU admission; concomitant and previous infusion of exogenous catecholamines were considered as exclusion criteria. Upper reference limits for serum norepinephrine, epinephrine and dopamine were 420 ng/mL, 84 ng/mL and 94 ng/mL respectively. We compared the serum catecholamine levels with biochemical markers of systemic inflammation, clinical data, and echocardiographic parameters of right and left ventricular dysfunction. A pilot study to verify the agreement on catecholamine blood withdrawal and analysis was performed. Transthoracic echocardiography was performed at ICU admission and presence of acute cardiac injury was assessed with high-sensitivity troponin I. Right ventricular (RV) systolic dysfunction was defined as tricuspid annular plane systolic excursion (TAPSE) < 1.7 cm, left ventricular (LV) systolic dysfunction was defined as LV ejection fraction (LVEF) < 50%. TAPSE was measured as the longitudinal systolic excursion of the tricuspid annulus plane from apical 4-chamber view. LVEF was measured using the biplane Simpson’s method.

The echocardiography was performed by board certified physicians who have previously underwent an intra-observer and inter-observer variability test for consistency and to limit methodological imaging interpretation bias [4].

The study was approved by the local ethical committee. The informed consent was obtained from the patients who survived and was waived in those who died in accordance with regulation in force during data collection. All the procedures were followed in accordance with the ethical standards of the responsible committee on human experimentation and with the Helsinki Declaration of 1975, as specified in the protocol.

SPSS 28.0.0 (IBM SPSS statistics) was used for data computation. Normal distribution of data was assessed with D’Agostino-Pearson test and histogram representation. Correlations were assessed with Pearson (standard deviation) or Spearman (interquartile range, IQR) tests depending on normal distribution. Logistic regression and Cox proportional hazards modelling were applied. For the Cox proportional hazards modelling, diagnostic plots for residuals and the Schoenfeld test proportional hazards was met, and these were found to be satisfactory. Values that were missing were assumed “missing at random”.

## 3. Results

Seventy-one patients with confirmed SARS-CoV-2 infection and moderate to severe ARDS were enrolled. The mean age was 62.2 (±11), 25.3% were female. The most frequent comorbidities were hypertension (55%), obesity (42.2%) and diabetes mellitus (21.1%). Eleven patients (15.5%) died during the admission in ICU; 11 patients (15.5%) were intubated within the first 6 h since ICU admission and 50% of overall patients received mechanical ventilation over ICU stay. Sixty-one patients (86%) required noradrenaline over the admission to maintain adequate perfusion pressure (mean arterial pressure ≥ 65 mmHg).

Table 1 shows the comparison of clinical features, echocardiographic parameters, biochemistry data, inflammatory markers, and serum catecholamine levels between alive and dead patients.

Median catecholamine levels were: norepinephrine 1886 ng/mL [IQR 994.25–3313.25], epinephrine 356 ng/mL [IQR 243.50–542.00] and dopamine 460 ng/mL [IQR 283.75–903.00]. Norepinephrine levels were higher in those with RV and LV systolic dysfunction (*p* < 0.001; r −0.541 and −0.510, respectively), higher C-reactive protein (CRP) (*p* < 0.001; r 0.451), higher interleukin (IL) 6 (*p* < 0.001; r 0.516). Both epinephrine and dopamine correlated with IL-6 (*p* < 0.01; r 0.31 and 0.301, respectively). However, they did not correlate with LVEF (*p* 0.0981 and 0.185, respectively), TAPSE (*p* 0.243 and 0.192, respectively), and CRP (*p* 0.095 and 0.159, respectively)

Univariable analysis by Cox proportional hazards regression modelling showed that age (1.13 [0.48–4.61]; *p* 0.035), norepinephrine (4.16 [1.85–5.13]; *p* < 0.001), LVEF < 50% (OR 2.22 [1.43–4.21]), IL-6 (OR 4.56 [2.0–5.52]), and CRP (2.08 [1.01–3.16]; *p* 0.002) had the highest risk of acute mortality. No correlation between risk of acute mortality, gender, comorbidities, body mass index and sequential organ failure assessment (SOFA) score was found in the univariable analysis (*p*-values > 0.1). Serum levels of dopamine and epinephrine (*p*-values > 0.5) as well as blood pressure and Horowitz index at ICU admission were not significantly correlated with the risk of acute mortality. Multivariable analysis showed that only norepinephrine (*p* 0.004; OR 2.85 [1.05–3.11]) and IL-6 (*p* 0.007; OR 1.37 [CI 95% 0.98–2.57]) retained in the model.

**Figure 1:**
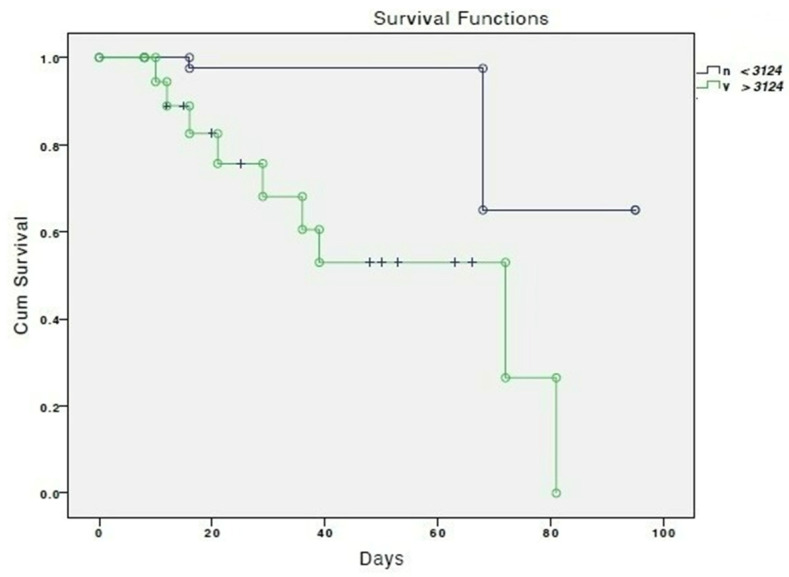
Cumulative survival according to serum norepinephrine levels lower (green line) and higher or equal (blue line) to 3124 ng/mL. “+” means censored.

Figure 1 shows the cumulative survival according to serum levels of norepinephrine.

## 4. Discussion

This is the first study proving the marked increase of blood catecholamines during the acute phase of critically ill COVID-19 and demonstrating their associations with inflammatory and clinical parameters.

COVID-19 is characterized in most severe forms by cytokine storm driven by hyperinflammatory state and uncontrolled immune system activation. Macrophage-derived cytokines as IL-6, IL-10, tumor necrosis factor-α, granulocyte colony stimulating factor and their downstream acute phase reactants (e.g., ferritin) are associated with greater disease severity and worse outcomes [3]. COVID-19 has been associated with various syndromes including: acute cardiac injury, takotsubo syndrome (TTS) [5], thromboembolism [6], severe hypoxemia and ventilatory/perfusion mismatch with loss of hypoxic vasoconstriction [7]. All of this are essentially characterized by increased inflammatory markers and organ-specific enzymes alterations [8]. In our cohort of acutely ill patients with moderate to severe COVID-19 ARDS, the marked increase in serum catecholamine levels (particularly norepinephrine) correlated with the degree of systemic inflammation, expressed by CRP and IL-6.

The complex interplay between sympathetic activation and inflammation has already been studied in completely different population than COVID-19 and ICU. High cortisol levels in cases of community-acquired pneumonia and COVID-19 patients were associated with increased mortality [1,9]. Catecholamines act on the hypothalamic-pituitary-adrenal axis affecting glucocorticoid-mediated immune signals influencing cortisol production and release. Conversely, significant increase of cortisol may potentially mitigate detrimental effect of catecholamines. Our findings may add further insight into the pathophysiological role of catecholamines in the acute phase of COVID-19 and they may reinforce the role of cytokine storm.

Catecholamines play a key role in the regulation of the cardiovascular, metabolic, and immune systems. Excessive endogenous (or exogenous) levels may result in diabetogenic states, severe cardiovascular dysfunction with or without tachyarrhythmias, hypercoagulability (thromboembolism), pulmonary (ventilation/perfusion mismatch and reverse compensatory hypoxic vasoconstriction) and immune dysregulation (cytokine storm).

The role of catecholamines in inducing cardiovascular dysfunction has been elucidated in the last two decades in the context of TTS. The main pathological mechanism is the hyperactivation of central autonomic nervous system leading to an enhanced sympathetic stimulation by endogenous catecholamine surge. This may mediate several effects on cardiovascular system. Epinephrine and norepinephrine play a direct effect on myocardium inducing the switch of G protein of the β-adrenergic receptors leading to ventricular function phenotypes (apical ballooning, inverted TTS, focal, and biventricular) and a vasoconstrictive effect on arterial vessels eventually leading to coronary micro- and macro-circulation dysfunction. Additionally, there is increasing evidence on the role of inflammation in the TTS acute phase, as identified by both increased levels of macrophage, IL-6, chemokines and by increased T2STIR signal at cardiac magnetic resonance [2,10].

The inverse correlation between serum norepinephrine levels and degree of RV/LV systolic impairment may suggest a detrimental role of autonomic nervous systemic potentially elicited by the inflammatory cascade on cardiac function related to COVID-19. As highlighted in the treatment bundle of the patients with TTS and hemodynamic instability [11], it should be explored whether the use of exogenous catecholamines in this sub-set of patients may lead to a reiteration of pathophysiological mechanism and further worsening cardiac function.

The main weakness of our study is the single measure of serum catecholamine levels within two hours since ICU admission. This analysis does not consider intraindividual variations in the dynamics of catecholamine response to stress and treatments as most of patients received catecholamines during the admission, therefore the results of blood samples would have been biased. The correlation with mortality after weeks of admission may have been affected by influencing factors and not only catecholamine at the time of ICU admission. Therefore, these results and eventual consequences on treatment should be confirmed in a larger validation study.

The role of inflammation in the severity of COVID-19, established since few months after the first outbreak, with prevention and blockade of cytokine storm have represented the focus of research efforts [12]. The clarification of the catecholamine role in the pathogenesis of different disease may play an important role in the consequent therapeutic decision pathway (for example, avoiding adrenergic catecholamine infusion) in patients with hemodynamic instability.

## Figures and Tables

**Table 1:** Comparison of clinical features, echocardiographic parameters, inflammatory markers, and serum catecholamine levels between alive and dead patients.

Data and Parameters	Overall Population (n = 71)	Alive (n = 60)	Dead (n = 11)
Males, n (%)	53 (74.6)	44 (73.3)	9 (81.1)
Age	62.2 ± 10.9	61.1 ± 11.0	68.4 ± 8.2
BMI, kg/m^2^	29.85 ± 5.95	29.45 ± 4.08	35.07 ± 16.12 ^§^
Diabetes mellitus, n (%)	16 (22.5)	15 (25)	1 (9.1)
Arterial hypertension, n (%)	39 (54.9)	33 (55)	6 (54.5)
Obesity, n (%)	30 (42.3)	25 (41.7)	5 (45.5)
CAD, n (%)	6 (8.4)	5 (8.3)	1 (9.1)
Chronic heart failure, n (%)	1 (1.4)	0 (0.0)	1 (9.1)
COPD, n (%)	4 (5.6)	4 (6.7)	0 (0.0)
CKD, n (%)	3 (4.2)	2 (3.3)	1 (9.1)
β-blockers, n (%)	17 (23.9)	12 (20)	5 (45.5)
ACEi, n (%)	21 (29.6)	18 (25)	3 (27.3)
Septic shock, n (%)	17 (23)	8 (13.3)	9 (81.8) *
Arrhythmias, n (%)	13 (18.3)	8 (13.3)	5 (45.5)
SOFA score	3.4 ± 1.1	3.3 ± 1.1	3.9 ± 1.0
HR, bpm	81.7 (±18.7)	82.6 (±19.4)	79.9 (±21.4)
SBP, mmHg	136.3 ± 22.8	136.0 ± 22.3	137.6 ± 26.8
MAP, mmHg	93.6 (±12.8)	94.3 (±12.7)	89.8 (±13.7) ^§^
Lactate, mmol/L	1.14 (±0.52)	2.80 (±0.48)	1.23 (±0.69) *
pH	7.39 ± 0.05	7.39 ± 0.05	7.38 ± 0.07
PaO_2_/FiO_2_, mmHg	152.7 ± 55.8	154.6 ± 58.7	141.2 ± 33.1
PaCO_2_, mmHg	36.3 ± 6.3	36.7 ± 5.9	36.0 ± 8.5
FiO_2_, %	68.0 ± 16.4	67.3 ± 16.8	72.0 ± 13.4
PEEP, cmH_2_O	11.6 ± 2.1	11.6 ± 2.1	12.0 ± 2.2
LVEF, %	50.4 (±7.9)	52.0 (±5.8)	42.1 (±11.6) ^§^
TAPSE, mm	17.9 (±1.9)	18.1 (±1.6)	16.9 (±3.3)
hs-TnI, ng/L	11.00 [4.75–34.75]	7.00 [4.00–25.75]	24.50 [8.00–207.00] ^¥^
Creatinine, mg/dL	0.80 [0.63–0.98]	0.75 [0.61–0.90]	1.18 [0.87–1.70] ^§^
PCT, ng/mL	0.16 [0.05–0.43]	0.15 [0.05–0.35]	0.46 [0.12–1.16]
CRP, mg/dL	9.92 [4.04–17.74]	8.23 [3.48–12.45]	27.80 [21.45–32.61] ^†^
WBC	8600 [5525–10,967]	8600 [5922–11,525]	7500 [4600–9800]
IL-6, pg/mL	45.70 [21.58–125.16]	37.88 [20.81–87.37]	171.90 [128.16–200.23] *
Norepinephrine, ng/mL	1886.00 [994.25–3313.25]	1721.50 [940.00–2810.50]	6152.00 [3614.75–9742.50] ^†^
Epinephrine, ng/mL	356.00 [243.50–542.00]	399.00 [254.00–553.50]	242.00 [197.75–489.75]
Dopamine, ng/mL	460.00 [283.75–903.00]	452.50 [266.50–776.50]	809.00 [335.25–1122.50]

Hemodynamic, hemogasanalysis and echocardiographic data are expressed as mean value (±standard deviation). Catecholamine levels, biochemistry parameters and inflammatory markers are expressed as median [interquartile range]. † *p*-value < 0.0001; * *p*-value < 0.001; ^§^ *p*-value < 0.01; ^¥^ *p*-value 0.02. Abbreviations: ACEi, angiotensin-converting-enzyme inhibitor; BMI, body mass index; CAD, coronary artery disease; CKD, chronic kidney disease; COPD, chronic obstructive pulmonary disease; CRP, C-reactive protein; FiO_2_, fraction of inspired oxygen; hs-TnI, high-sensitivity troponin I; HR, heart rate; IL-6, interleukin 6; LVEF, left ventricular ejection fraction; MAP, mean arterial pressure; PaCO_2_, partial pressure of carbon dioxide in arterial blood; PaO_2_, partial pressure of oxygen in arterial blood; PCT, procalcitonin; PEEP, positive end-expiratory pressure; SBP, systolic blood pressure; TAPSE, tricuspid annular plane systolic excursion; WBC, white blood cell.

## Data Availability

The data presented in this study are available on reasonable request from the corresponding author.
